# Students’ perception and learning experience in the first medical clerkship

**DOI:** 10.1186/s12909-022-03754-4

**Published:** 2022-09-27

**Authors:** Marc Gottschalk, Christian Albert, Katrin Werwick, Anke Spura, Ruediger C. Braun-Dullaeus, Philipp Stieger

**Affiliations:** 1 University Medicine Magdeburg, Center for Internal Medicine, University Clinic for Cardiology and Angiology, Leipziger Str. 44, D-, 39120 Magdeburg, Germany; 2 Diaverum Renal Services, MVZ, Potsdam, Germany; 3 Student Affairs, Medical Faculty, Magdeburg University, Magdeburg, Germany; 4 Federal Centre for Health Education, Cologne, Germany

**Keywords:** Teaching and learning, Work-based, Clinical, Medical clerkship, Students’ perception, Medical education

## Abstract

**Background:**

The German clerkship (“Famulatur”) is the first phase in medical education, in which students learn from a physician’s perspective. According to the German Licensing Regulations for Physicians, students shall “familiarise” with providing care. However, specific learning objectives for the clerkship are not defined, although the acquisition of different competencies is implicitly demanded. Therefore, an additional understanding of the clerkship students’ learning experience is needed. The goal of this study is to explore the student’s learning perspective and experiences in the clerkship.

**Methods:**

Twelve guideline-based interviews were conducted with third year medical students. All participants completed their first clerkship. A qualitative content analysis was performed. The inductively identified categories were transferred into a quantitative questionnaire using a 5-point Likert-scale to explore their relevance in a validation cohort. The questionnaire was completed by 222 clinical students of the Otto-von-Guericke-Universität Magdeburg.

**Results:**

The qualitative analysis led to 26 individual items assigned to 4 main categories that describe the clerkship experience: 1) “coping with insecurities”, 2) “the clerkship as a social arrangement”, 3) “the clerkship as a learning opportunity” and 4) “the clerkship as a teaching opportunity”. In the quantitative validation cohort, category one yielded a well-balanced result (median 3 = “neither agree nor disagree”; IQR 2–4), items addressed in categories 2–4 were generally supported by the students, predominantly selecting “strongly agree” or “agree” (Median 2; IQR 1–2 for each category). Students rated the role of the clinical team as especially important for their learning success and feared exclusion or negative reactions.

**Conclusions:**

The medical clerkship provides an institutional, professional, and social framework, in which students are learning. Insecurities arose from curricular inconsistencies, a high dependency on the clinical team as well as the absence of specific learning objectives. Therefore, a better curricular integration regarding the semester structure and the learning objectives of the German clerkship is needed.

**Supplementary Information:**

The online version contains supplementary material available at 10.1186/s12909-022-03754-4.

## Background

Medical clerkships in Germany (‘Famulatur’) provide the first opportunity in medical education in which students learn practically from a physician’s perspective [1]. According to the German licensing regulations for Physicians (“Ärztliche Approbationsordnung”), the clerkships’ purpose is to familiarise medical students with providing health care in outpatient and inpatient care facilities [1]. The completion of 4 months of medical clerkship is mandatory [1]. In Germany however, the medical clerkship is not regulated with individual learning objectives, but is intended to promote the development of individual practical skills, knowledge and professionalisation [2–4]. Accordingly, little is known about students’ individual educational processes and associated learning success in the medical clerkship [4] . Previously, German reports issuing medical clerkships were focussed on the evaluation of teaching interventions [2, 3, 5]. Medical Clerkship students’ competencies gained in the field of professionality, interprofessionality, or learning processes were rarely addressed in German literature [4, 6].

Surmon et al. [7] identified key factors for medical clerkship preparation and experience, which were partly modifiable like faculty curricula, individual competency, workload or being part of the team. Others [8] focussed on the role of professionalism in medical education, especially in internships, or promoted a more stress-oriented perspective [9]. However, insight on students’ perceptions and learning experience in the clerkship is important, not only for current health professions education context, but also with regard to recent changes in German licensing regulations [10, 11]. Therefore, educational research, that is epistemically based on sociological and phenomenological approaches, is needed [4]. Accordingly, the aim of this study was to investigate the students’ perspective and their learning experiences in the clerkship using qualitative content analysis of guided interviews and subsequent quantitative evaluation in an independent student cohort.

We hypothesise that this study reveals new insights into students’ insecurities and problems facing the first-time exposure to clinical practice as well as insights on individual learning behaviour and challenges [4, 6].

## Methods

A qualitative approach using a guideline based on international literature was chosen as recommended by Steinke and Reimann to achieve a deeper understanding of students’ clerkship experiences [4, 12]. The qualitative approach was used to identify relevant categories for students’ medical clerkship experiences. A content analysis in accordance with Mayring [13] was performed using guided single person interviews. The participants of this derivation cohort were third year - medical students of Magdeburg University, just having passed their first medical clerkship. To facilitate and improve the recruiting process a modified snowball system was used, that takes advantage of inviting study participants by direct appeal based on the recommendations of other participants [14]. Twelve medical students were selected, in order to represent a maximal diversity concerning gender, speciality and site of medical clerkship. After consent and beginning of the interview, there were no interview abortions or withdrawals by the participants. Data acquisition was halted, when additional interviews did not add new categories and thus theoretical saturation was reached [13]. Participant feedback on material and outcomes was not obtained.

Subsequently, based on the results of the qualitative content analysis, a quantitative questionnaire using Likert scales was created. Thus within a prospective validation study, the questionnaire was handed out to a validation cohort of 222 medical students (third to fifth year, independently of the first cohort) of the same faculty, who had passed at least one medical clerkship. Students were surveyed during curricular presence courses (e.g. microscopy) in the third and fifth year. They were informed about the option to take part voluntarily and extra time was provided for the completion of the questionnaire. The study was approved by the Institutional review board of the Otto-von-Guericke University Medical Faculty Ethics Committee, Magdeburg, Germany (Case no.: 65/15). The study protocol was in concordance with the ethical guidelines of the Declaration of Helsinki. Informed consent was obtained from all participants.

### Qualitative assessment in the derivation cohort

We performed guided single person interviews in the derivation cohort in reference to themes previously identified of value for students learning success in medical education such as interprofessionality, case-specific and system-specific reference, practical skills as well as expectations [3].

**Fig. 1:**
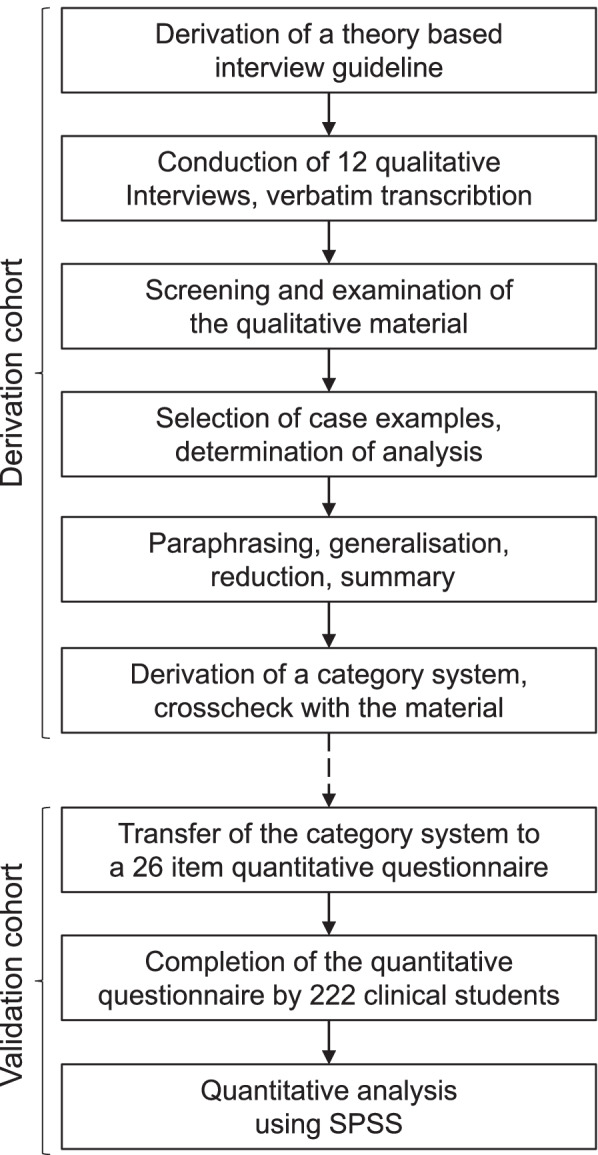
Flow-Chart of the study

Interviews for a qualitative assessment were performed face-to-face on the campus of Otto-von-Guericke-University Magdeburg. No field notes were taken. Recordings of the interviews were transcribed and anonymised [14, 15]. To identify the different citations, each interview was given an anonymised code. A qualitative content analysis in accordance to Mayring [13] was performed: In a first step five exemplary cases were selected and used for inductive categorisation. These were further discriminated into selected items, which were afterwards validated on the remaining material (Fig. 1). The survey was performed in German. On the selected sample material, we used forward and backward translation to ensure semantic appropriate English translations of the content. No specific software solution was used for the analysis. An interdisciplinary research workshop discussing and interpreting qualitative key passages on an interdisciplinary basis was used including researchers with medical, psychological and social science backgrounds, as proposed by Steinke et al. [12]. Conducting the analysis, the COREQ (COnsolidated criteria for REporting Qualitative research) checklist was applied [16]. The appropriate checklist is available as Supplemental Table 4.

### Quantitative evaluation of category items in the validation cohort

Subsequently, categories and subcategories, identified in the derivation cohort, were reassessed in the validation cohort. In accordance with Brod et.al [17] and Lasch et al. [18] literature review, interviews and subsequent qualitative analysis were performed to generate relevant items for the quantitative analysis. The quantitative questionnaire was based on the qualitative categories identified in the derivation cohort (Supplemental Table 3). Cognitive debriefing interviews to potentially enhance content validity of the quantitative questionnaire [17] were not performed. In the questionnaire, practical skills were classified into hard skills and soft skills [19]. Students’ perceptions were measured and evaluated based on a 5- point Likert scale [20]. Participants responded to statements concerning their expectations and clerkship experiences on a scale from ‘strongly agree’ to ‘strongly disagree’ (1 = Strongly Agree, 2 = Agree, 3 = Neither agree nor disagree, 4 = Disagree, 5 = Strongly disagree). Data were described using frequency distributions, measurements of central tendencies and dispersion measures. Histograms were used for testing data for normal distribution. An exploratory factor analysis (extraction via maximum likelihood) was not performed, because the data had a Kayser-Meyer-Olkin coefficient of 0.65. The original questionnaire was written in German. We used SPSS Statistics version 26 (IBM Corp., Armonk, New York, USA).

## Results

### Qualitative assessment

In the derivation cohort, 12 interviews were performed. The cohort consisted of 66% female participants, the mean age was 23,25 years (median 22,00 a; SD 3,02 a). Working experiences in the health care sector (e.g. voluntary civilian service, nursing) were present in 42% of the participants. Regarding the different disciplines, 33% of the participants conducted their recent clerkship in internal medicine, 25% in surgical disciplines, 25% at general practitioners and 17% in other disciplines. More than half of the clerkships were conducted at university hospitals (58%). Through the qualitative content analysis four inductive categories could be identified: ‘coping with insecurities’, ‘medical clerkship as a social arrangement’, ‘medical clerkship as a learning opportunity ‘and ‘medical clerkship as a teaching opportunity’. Since the interviews were originally performed in German, categories, items and anchor quotes are provided in the original German version and corresponding English translation in Supplemental Tables 1 and 2.

#### Coping with insecurities

A central motive analysing students’ perception is ‘coping with insecurities’. Triggers for those are manifold, and were described by the following subcategories:

- Knowledge and skills between theory and practice
- Error culture
- Role insecurity
- Clerkship as a permanent challenge for the staff
- Inappropriate behaviour

Students often experienced clerkship-situations in which they felt unprepared for specific skills or knowledge demanded. Reasons in many cases were a lack of training and curricular inconsistencies. A typical example is the daily routine of obtaining blood samples:

At first, I was a bit surprised. I told them, that I have done it only on an anatomic model and that I might fail […]. *(L2, line 56 - 57)*

Students also anticipated a rigid error culture, which was described as stigmatising and degrading. This can be explicitly shown in linguistic motives like ‘finish off’ (L2, line 104) or ‘being slagged’ (L3, line 76). Furthermore, in the clinical setting, students have to deal with their new tasks and responsibilities. The clerkship is also a challenge for the medical staff because they have to adapt to - and integrate new students, repeatedly. The students themselves, their personality, abilities and skills are -at first- unknown to them. Also, unprofessional behaviour is a problem in clerkships, leading to insecurities and stress. For example, excessive joking was experienced as inappropriate by the students:

The team was so uninvolved. I found it somehow scary. They made jokes all the time, but I could not identify serious working or conscientious working. (L3, line 90-93)

#### The clerkship as a social arrangement

Getting involved and integrated further into in the ward team is another important factor, clerkship students need to cope with. This was described by the following subcategories:

- Relationship to the physicians
- Clerkship in an interprofessional setting
- Disturbances of relationship and role expectations

The relationship to the physicians as direct supervisors seems especially important at the beginning of a clerkship. For example, the student in interview L1 describes how he got introduced on his first day:

On the first day, he took us upstairs […]. We were introduced to the other physicians, the chief of department, the residents. It was very nice. (L1, line 70-74)

Also, students reflected their relationship to other health care professionals before and after the clerkship. Furthermore, factors like temporal or personal shortages were identified as triggers for disturbances concerning team integration and role expectation. For example, one student tolerated being tasked with excessive blood withdrawal instead of taking part in the grand rounds, because he identified with his residents:

In the end, I could have said no, I don’t do that. I realised how busy they were and that they worked overtime most days […]. (L6, line 52-55)

#### The clerkship as a learning opportunity

Students regarded the clerkship primarily as a learning opportunity, that was described by the following subcategories:

Influencing factors concerning the learning behaviour

- Self-assessment and planning of individual learning objectives
- Clerkship evaluation basing on learning objectives
- Influence factors for choosing a specific clerkship

Learning processes

- Experiencing central procedures and actors
- Getting a broad view
- Soft skills and patient interaction
- Training of hard skills
- Learning general routines

Before choosing a clerkship, students analysed their competencies and selected ‘strategic’ (L1 line 306) learning objectives. They evaluated their clerkships’ success based on these issues. For example, the student in interview L10 regretted, that he was not able to perform enough practical activities:

I wanted to do more hands-on medicine. As of yet, I only did some auscultation, but that’s it regarding practical activities […] (L10 line 50 - 51).

Regarding the choice of a specific clerkship, students applied criteria such as personal interest or the wish to gain insights into potential future specialisation, as reported in the interview L6:

I really wanted to pursue internal medicine, because I am personally not interested in surgery at the moment […] (L6, line 103-104)

Also, experiencing diagnostic and therapeutic procedures was a prominent learning objective. Students wished to be trained in practical skills such as blood withdrawal or the performing of clinical examinations. Students argued, that these objectives depict ‘basic skills’ (L2, line 72). Furthermore, students wished to familiarise themselves with daily routines such as grand rounds. Exemplary, the student in the interview L2 describes that he wanted to ‘experience the everyday ward life’ (L2, line 313–315).

#### The medical clerkship as a teaching opportunity

If clerkships are considered learning opportunities, they may also be considered teaching opportunities. Two main categories of teaching were identified in the material:

Teaching activities

- Show
- Explain
- Instruct
- Oversee
- Supervise
- Participate in decision making

Concepts of clerkship training

- Introduction to the clerkship
- Knowledge transfer through key characters
- Interprofessional learning
- Missing of training concepts

Students learned through a variety of activities ranging from simple ones like ‘being shown something’ to more laborious activities like ‘being supervised’. Demonstrations were common in many clerkships, for example in the interview L4:

He showed me the coronary arteries in the catheterisation laboratory. If there was a stenosis, he precisely pointed on it […]. (L6, line 81-83)

In most of the interviews, explanations and explications were described. On a more practical level students were instructed regularly. When performing invasive tasks, a supervision by physicians was necessary, needing additional temporal resources. Another important learning activity, that was only seldomly experienced is the integration of students’ opinion and ideas in medical decision processes. In many cases, experienced by the students, they were not asked for their diagnostic and/or therapeutic reasoning to further develop a patient’s case. The material also included concepts consisting of multiple of the above-mentioned learning activities. Furthermore, medical education in clerkships was experienced as an interprofessional task, in which students for example benefited from the commitment of surgical nurses:

The nurses in the operating theatre really wanted to show things to us and tried to guide us […]. (L3, line 294-295)

Also, there were reports about instruction and orientation procedures at the beginning of the clerkship. In the interview L1 students were shown the facilities and where to find the different working materials:

When we arrived at the clinic the first day, the ward assistant gave us an introduction. She showed us around. A nurse explained to us how we’d get our equipment for our daily activities […] (L1 line 27-30)

The transfer of knowledge and training of skills was primarily performed by key characters. For example, a senior physician first demonstrated a cardiac defect by using an anatomical model and subsequently transferred the case to his everyday work. However, if educational training concepts were neglected, this negatively influenced students’ learning success.

### Quantitative validation of category items

**Fig. 2:**
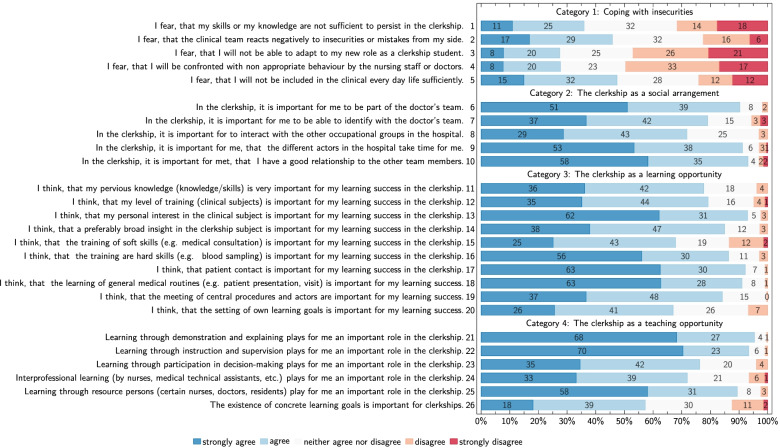
Quantitative survey results in the validation cohort in percent (%) on a 5-level Likert scale

Based on the results of the qualitative assessment in the derivation cohort, a 26-item questionnaire using Likert scales was prepared for quantitative assessment (Fig. 2, Supplemental Table 3).

We received high agreement ratings for nearly all items in the four categories: ‘Coping with insecurities’, ‘the clerkship as a social arrangement’, ‘the clerkship as a learning opportunity’ and ‘the clerkship as a teaching opportunity’. Quantitative survey results assessing the relevance of category items to students in the validation cohort using a five-level Likert scale are provided in Fig. 2 and Supplemental Table 3.

‘Coping with insecurities’ was the most heterogeneous and least agreed category (Fig. 2), suggesting a more diverse clerkship experiences in this field. The biggest fear of the students was negative reactions from the clinical team to mistakes or insecurities (45% strongly agree or agree; mean 2,66; SD 1,13). It was followed by the worry of segregation by the team (47% strongly agree or agree; mean 2,74; SD 1,22). Closely connected is the category ‘the clerkship as a social arrangement’, having continuously high agreement rates. Most important for the students was a good relationship to all other members of the clinical team (93% strongly agree or agree; mean 1,59; SD 0,76). Also, students wished, that the different team members should take time for them (91% strongly agree or agree; mean 1,53; SD 0,78). Our results indicate that the students primarily identified with the physician team but were at the same time interested in the interaction with other occupational groups.

In the category ‘the clerkship as a learning opportunity’ students rated patient contact as most important for their learning success (92% strongly agree or agree; mean 1,46; SD 0,69). It was closely followed by the learning of general medical routines (91% strongly agree or agree; mean 1,47; SD 0,67) and the personal interest in the clerkship speciality (93% strongly agree or agree; mean 1,47; SD 0,70). Soft-skills and self-directed learning were rated less important.

With regards to ‘the clerkship as a teaching opportunity’ students rated explanations and demonstrations (95% strongly agree or agree; mean 1,37; SD 0,59) as well as instructions and supervision (93% strongly agree or agree; mean 1,37; SD 0,64) as most important for their learning process. Participation in decision making was less important for the students. They confirmed that key persons (e.g. specific doctors or nurses) are important for their learning experience (89% strongly agree or agree; mean 1,55; SD 0,75). Interprofessional learning and the existence of clearly defined learning objectives was seen as less important.

## Discussion

We assessed students’ perception and learning experience in the first medical clerkship using guideline-based qualitative interviews. Students’ perception was summarised in 26 statement items, assorted to four main categories: ‘coping with insecurities’, ‘the clerkship as a social arrangement’, ‘the clerkship as a learning opportunity’ and ‘the clerkship as a teaching opportunity’. Subsequently, these statement items were quantitatively assessed for relevance in a validation cohort of 222 clinical students.

We found, that the clerkship may be characterised as a complex social and educational phase: Students rated patient contact and general routines to be essential for their learning success (92 and 91% strongly agree or agree, respectively). To achieve enough learning opportunities students focussed on becoming a part of the ward team (93% strongly agree or agree). They feared exclusion (47% strongly agree or agree) and negative reactions to insecurities (45% strongly agree or agree), which would lead to disadvantages in their learning process.

Surmon et al. [7] identified relevant factors for medical clerkship experience like faculty curricula, individual competency or being part of the team. The clerkship is described as an important, but difficult, transitional phase [21]. Students are confronted with stress and high workload in their process of medical professionalisation [22, 23]. In concordance with our findings, feelings of insufficiency and insecurity among students are described, when competencies and expectations do not align [24]. Furthermore individual characteristics like gender [25, 26] and prior work experience [27] may influence the clerkship experience.

Since previous studies [2, 5] focused on single aspects of the medical clerkship such as learning activities, professionalism, or social influence factors, there is a lack of literature investigating on their interaction [4].

Our findings may suggest that the learning behaviour of students is influenced by their prior knowledge and interests. This shows similarities to the concept of ‘self-directed learning’ [28, 29]. The present findings highlight individualised learning processes such as self-assessment and evaluation of learning progress, that were previously described by O’Brian et al. [30] and Hauer et al. [31]. However, students tended to entrust the organisation of learning processes in the clerkship to their teachers [32, 33]. As a consequence, many learning processes are dependent on the time and resources physicians and other health care professionals allocated to their students [34] as well as their teaching behaviour [35, 36]. Moreover, our findings acknowledge, that interprofessional learning experience fulfils an important role in the students’ clerkship experience, but these learning encounters were often not structured nor were clear learning objectives available [3]. Also, the way physicians and other members of the ward staff practiced professionality played an important role [8]. Reimer et al. [37] reported on pre-clerkship students’ perception of medical professionalism and how it changed over time. Thus our study concludes, that the medical clerkship can be seen as a social, institutional and professional arrangement: Factors like ward organisation [4, 8, 34], the daily interaction with patients and other healthcare professionals, as well as the handling of different learning processes lead to the creation of an individual system of relationships and values shaping the students medical identity [8].

Our findings suggest that medical clerkships may be regarded as social, institutional, and professional arrangements, in which students transition from theory based medical education to practice based learning. This study emphasizes a focus on insecurities during the clerkship by disclosing, that a significant part of the students fear negative reactions to uncertainties or mistakes by the ward team (45% strongly agree or agree). Attention of medical trainers should be focussed on this issue, that may be caused by curricular inconsistencies, a perceived lack of skills and knowledge [21, 38], as well as the absence of specialised preparation courses [3]. The high degree of inconsistency in our sample regarding insecurities might arise from the different varying degrees of competency between the third and fifth year of medical education [11, 39]. Also, the influence of personal characteristics seems possible [25]. Furthermore, our findings highlight the importance of team integration (93% strongly agree or agree) as described by Hauer et al. [31]. Medical educators have to acknowledge, that the clerkship experience is influenced by the social and organisational structures of the clerkship site, which can promote or hinder effective learning [4]. Therefore, didactic concepts, that facilitate the integration of students may be indicated. Additionally, our findings underline the importance of learning clinical routines (91% strongly agree or agree) and patient contact (92% strongly agree or agree). Although, students seem to have developed their own learning objectives, it is not clear if those verily augment students’ clinical competencies. A catalogue of predefined learning objectives as for example proposed by Jerg et al. [40] may help students to strengthen their competence profile. Finally, we found high relevance of ‘learning by key characters’ (89% strongly agree or agree), which is in line with previous findings [35, 41] and thus further stresses the importance didactic training for health care professionals.

One of the major strengths of the qualitative design is the open approach that enables the formation of new hypothesis and models [42]. An obvious limitation is the small number of interviews and method of recruitment, that is however typical for qualitative approaches [12]. We therefore evaluated our findings quantitatively using Likert scale questionnaires in a larger validation cohort. Not all students answered all of the survey’s questions. However, response rates were generally high (84 to 91%, Supplemental Table 3). Also, a limiting factor is unavailability of further epidemiological information on the quantitative dataset. Another limitation is the focus on students’ experiences, leaving aside factors such as individual characteristics and abilities, although such confounding may be reasonable [43, 44]. Further examination of these factors may improve the understanding of students’ learning approach and attribution of personal goals in the medical clerkship.

In the absence of curricular defined learning objectives, medical students seem to have developed their own perceptions and learning objectives regarding the clerkship. Inconsistencies and incongruences of the clerkships’ potential as a learning environment [45] promoted the development of preparational courses to support students learning efforts [3], that could be further optimized to considering the present findings. In addition, the German medical clerkship may demand for a better curricular integration regarding temporal and thematic aspects, a clear differentiation from later practical phases, as well as a definition of its specific learning objectives [1, 11, 46], specifically in light of recent changes in German licensing regulations [10, 11].

## Conclusion

The identified categories ‘coping with insecurities’, ‘the clerkship as a social arrangement’, ‘the clerkship as a learning opportunity’, ‘the clerkship as a teaching opportunity’, and their associated items are relevant to students and their learning process. Our findings underline that the medical clerkship provides an institutional, professional and social framework, in which students are learning. Insecurities arose from curricular inconsistencies, the absence of specific learning objectives, and a high dependency on the medical staff. We, therefore, call for a better curricular integration as well as a clear definition of learning objectives for the German medical clerkship.

## Supplementary Information

**Additional file 1: Supplemental Table 1.** Categories and items in English translation and original German version. **Supplemental Table 2.** Extracts from the original German interviews (anchor quotations), referred to in the results section. **Supplemental Table 3.** Quantitative survey results from the validation cohort evaluated on a five-level Likert scale.

**Additional file 2: Supplemental Table 4.** COREQ checklist.

## Data Availability

The qualitative data recorded for the study may not be shared publicly in order to ensure participants’ privacy protection. The quantitative datasets generated and/or analyzed during the current study are not publicly accessible but available from the corresponding author upon reasonable request.
