# Supplementary material for: Students’ perception and learning experience in the first medical clerkship
Source: BMC Med Educ. 2022 Sep 27;22:694. doi: 10.1186/s12909-022-03754-4 (PMC9513910; doi:10.1186/s12909-022-03754-4)
Supplement: Supplementary file 1 — Additional file 1: Supplemental Table 1. Categories and items in English translation and original German version. Supplemental Table 2. Extracts from the original German interviews (anchor quotations), referred to in the results section. Supplemental Table 3. Quantitative survey results from the validation cohort evaluated on a five-level Likert scale. [file 12909_2022_3754_MOESM1_ESM.docx]

**Students’ Perception and learning Experience in the first medical Clerkship**

Marc Gottschalk^a*^, Christian Albert^a,b*^, Katrin Werwick^c^, Anke Spura^d^, Ruediger C. Braun-Dullaeus^a^, Philipp Stieger^a,e^

*^a^University Medicine Magdeburg, Center for Internal Medicine, University Clinic for Cardiology and Angiology, Magdeburg, Germany; ^b^Diaverum Renal Services, MVZ Potsdam, Germany; ^c^Deans Office of Student Affairs, Medical Faculty, Magdeburg University, Germany; ^d^Federal Center for Health Education, Cologne, Germany; ^e^Center for Vascular Medicine and Interventional Angiology, Mainz, Germany*

*contributed equally to the study

**Corresponding Author**

Dr. med. Marc Gottschalk

University Medicine Magdeburg

University Clinic for Cardiology and Angiology

Leipziger Str. 44

D- 39120 Magdeburg

marc.gottschalk@med.ovgu.de

| **Supplemental Table 1.** Categories and items in English translation and original German version |  |
| --- | --- |
|  |  |
| **Categories and items in English translation** | **Categories and items in original German version** |
|  |  |
|  |  |
| **Category 1: Insecurities in the Clerkship** | **Kategorie 1: Unsicherheiten in der Famulatur** |
| 1. I fear, that my skills or my knowledge are not sufficient to persist in the clerkship. | 1. Ich habe Angst davor, dass meine Fähigkeiten bzw. mein Wissen nicht ausreichen, um im Famulaturalltag zu bestehen. |
| 2. I fear, that the clinical team reacts negatively to insecurities or mistakes from my side. | 2. Ich habe Angst davor, dass das Klinikteam negativ auf Unsicherheiten oder Fehler meinerseits reagiert. |
| 3. I fear, that I will not be able to adapt to my new role as a clerkship student. | 3. Ich habe Angst davor, dass ich mich nicht in meine Rolle als Famulant hineinfinde. |
| 4. I fear, that I will be confronted with non appropriate behavior by the nursing staff or doctors. | 4. Ich habe Angst davor, dass ich mit Fehlverhalten durch Pflegepersonal oder Ärzte konfrontiert werde. |
| 5. I fear, that I will not be included in the clinical everyday life sufficiently. | 5. Ich habe Angst davor, dass ich nicht hinreichend in den Klinikalltag einbezogen werde. |
|  |  |
| **Category 2: The clerkship as a social arrangement** | **Kategorie 2: Die Famulatur als soziales Arrangement** |
| 6. In the clerkship it is important for me to be part of the doctor's team. | 6. In der Famulatur ist mir wichtig, Teil des ärztlichen Teams zu sein. |
| 7. In the clerkship it is important for me to be able to identify with the doctor's team. | 7. In der Famulatur ist mir wichtig, mich mit dem ärztlichen Team identifizieren zu können. |
| 8. In the clerkship it is important for me to interact with the other occupational groups in the hospital. | 8. In der Famulatur ist mir wichtig, auch mit anderen Berufsgruppen im Krankenhaus zu interagieren. |
| 9. In the clerkship it is important for me, that the different actors in the hospital take time for me. | 9. In der Famulatur ist mir wichtig, dass sich die verschiedenen Akteure im Krankenhaus Zeit für mich nehmen. |
| 10. In the clerkship it is important for me, that I have a good relationship to the other team members. | 10. In der Famulatur ist mir wichtig, eine gute Beziehung zu den anderen Teammitgliedern zu haben. |
|  |  |
| **Category 3: The clerkship as a learning opportunity** | **Kategorie 3: Die Famulatur als Lernchance** |
| 11. I think, that my pervious knowledge (knowledge/skills) is very important for my learning success in the clerkship. | 11. Ich denke, dass meine Vorkenntnisse (Wissen/Fertigkeiten) für meinen Lernerfolg in der Famulatur sehr wichtig sind. |
| 12. I think, that my level of training (clinical subjects) is important for my learning success in the clerkship. | 12.  Ich denke, dass mein Ausbildungsstand (klin. Fächer) wichtig für meinen Lernerfolg in der Famulatur ist. |
| 13. I think, that my personal interest in the clinical subject is important for my learning success in the clerkship. | 13. Ich denke, dass mein persönliches Interesse am Famulaturfach für meinen Lernerfolg in der Famulatur wichtig ist. |
| 14. I think, that a preferably broad insight in the clerkship subject is important for my learning success in the clerkship. | 14. Ich denke, dass ein möglichst breiter Einblick in das Famulaturfach für meinen Lernerfolg in der Famulatur wichtig ist. |
| 15. I think, that the training of soft skills (e.g. medical consultation) is important for my learning success in the clerkship. | 15. Ich denke, dass das Training von Softskills (z.B. ärztliche Gesprächsführung) für meinen Lernerfolg in der Famulatur wichtig ist. |
| 16. I think, that  the training hard skills (e.g. blood sampling) is important for my learning success in the clerkship. | 16. Ich denke, dass das Training von Hardskills (z.B. Blutentnahme) für meinen Lernerfolg in der Famulatur wichtig ist. |
| 17. I think, that patient contact is important for my learning success in the clerkship. | 17. Ich denke, dass Patientenkontakt für meinen Lernerfolg in der Famulatur wichtig ist. |
| 18. I think, that the learning of general medical routines (e.g. patient presentation) is important for my learning success. | 18. Ich denke, dass das Erlernen allgemeiner ärztlicher Routinen (z.B. Patientenvorstellung, Visite) für meinen Lernerfolg in der Famulatur ist wichtig. |
| 19. I think, that the meeting of central procedures and actors are important for my learning success. | 19. Ich denke, dass das Kennenlernen zentraler Prozeduren und Akteure für meinen Lernerfolg in der Famulatur wichtig ist. |
| 20. I think, that the setting of own learning goals is important for my learning success. | 20. Ich denke, dass das Setzen eigener Lernziele für meinen Lernerfolg in der Famulatur wichtig ist. |
|  |  |
| **Category 4: The clerkship as a teaching opportunity** | **Kategorie 4: Die Famulatur als Lehrchance** |
| 21. Learning through demonstration and explaining plays for me an important role in the clerkship. | 21. Für mich spielt in der Famulatur das Lernen durch Zeigen und Erläutern eine wichtige Rolle. |
| 22. Learning through instruction and supervision plays for me an important role in the clerkship. | 22. Für mich spielt in der Famulatur das Lernen durch Anleiten und Supervidieren eine wichtige Rolle. |
| 23. Learning through participation in decision-making plays for me an important role in the clerkship. | 23. Für mich spielt in der Famulatur das Lernen durch Beteiligung an Entscheidungsfindungen eine wichtige Rolle. |
| 24. Interprofessional learning (by nurses, medical technical assistants) plays for me an important role in the clerkship. | 24. Für mich spielt in der Famulatur interprofessionelles Lernen (durch Pflegekräfte, MTAs, etc.) eine wichtige Rolle. |
| 25. Learning through resource persons (certain nurses, doctors, residents) play for me an important role in the clerkship. | 25. Für mich spielt in der Famulatur das Lernen mit Hilfe von Bezugspersonen (bestimmte Pflegekräfte, Stationsärzte, Oberärzte) eine wichtige Rolle. |
| 26. The existence of concrete learning goals is important for clerkships. | 26. Mir ist das Vorhandensein konkreter Lernziele für Famulanten wichtig. |
|  |  |

| **Supplemental Table 2.** Extracts from the original German interviews (anchor quotations), referred to in the results section. | | |
| --- | --- | --- |
|  |  |  |
| **Category** | **Item** | **Anchor quotations** |
|  |  |  |
|  |  |  |
| **#1**  **Unsicherheiten in der Famulatur** | **Ungleichzeitigkeiten zwischen Theorie und Praxis** | „[…] aber jetzt vor allem Medikamentenfragen und Therapiefragen oder spezifische Krankheitsbilder, auch EKG war schwierig, weil mans ja noch nicht so gehabt hat, EKG die Physiologiekenntnisse, die waren eher begrenzt, da konnte man nicht so viel mit anfangen […]" (L2 Zeile 100 - 104) |
|  | **Fehlerkultur** | „Also wir wollten zusammen machen, und wir wollten auf ne Station gehen, die, wo wir wissen, dass die relativ nett sind, weil wir hatten son bisschen Angst. [...] dass die uns anmachen [lacht]" (L3, Z 265 - 269) |
|  | **Rollenunsicherheit Famulierender** | „Ja des war irgendwie etwas ungewohnt, weil ich noch gedacht hab so für mich... seh mich noch nicht so wirklich als fertiger Arzt. Ich bin jetzt im klinischen Teil, es macht total Spaß und is super, aber es ist noch nicht so, dass sie mich deswegen alle siezen müssen und des sowas außergewöhnliches ist, dass ich Medizinstudent bin und deswegen hats mich am Anfang son bisschen gestört." (L4 Zeile 148 - 152) |
|  | **Famulatur als ständige Herausforderung für das Personal auf Station** | „[…] ähm also am Anfang muss man natürlich sagen, ich glaube aber das hat man auf den meisten Famulaturstationen, wenn man den ersten Tag da ist, ist man erstmal nen bisschen überfordert und die Leute wissen tatsächlich noch nicht so recht, wie sie mit einem umgehen sollen […]“ (L1 Zeile 24 - 26) |
|  | **Unethisches Verhalten** | „Weiß ich nicht, so unbeteiligt, das fand ich irgendwie erschreckend und dann Witze gemacht ja aber so ernsthaftes Arbeiten und gewissenhaftes Arbeiten, was auch zielführend ist, hab ich einfach nicht erkannt. Ich fand das so larifari alles und nich, absolut nich ernsthaft. Die haben unheimlich die ganze Zeit Witze gerissen, es war wirklich sehr lustig, aber die Ernsthaftigkeit hat dort wirklich gefehlt […]“ (L3 Zeile 90 - 94) |
| **#2**  **Famulatur als soziales Arrangement** | **Die Beziehung im und zum ärztlichen Team** | „[…] oben is, is gerad chirurgische Besprechung, Fallbesprechung und wir sollen mitkommen und dann hat er uns tatsächlich am ersten Tag direkt nach Blutabnehmen mit hoch genommen und hat auch uns da auf ne Patientenliege gesetzt oben bei der Besprechung und uns auch vorgestellt den Ärzten oben, also dem Chef, den Oberärzten, also es war sehr nett [...]" (L1 Zeile 70 - 74) |
|  | **Famulatur in einem interprofessionellen Setting** | „Ich hatte nich erwartet, dass das Verhältnis zwischen den Schwestern und den, äh, Ärzten oder den Assistenzärzten die waren, dass es so gut ist, da gibts ja das Klischee, dass da immer sone bisschen sone Fachunterschied ist, das hab ich so überhaupt nicht wahrgenommen." (L2 Zeile 87 - 89) |
|  | **Störungen von Beziehungs- und Rollenerwartungen** | „Ja letztendlich, ähm, hätte ich wahrscheinlich auch sagen können, ne ich mach das nicht, aber ich hab eben schon auch gesehen, wie die im Stress sind und dass die die meisten Tage Überstunden sowieso schon machen [...]" (L6 Zeile 52 - 55) |
| **#3**  **Famulatur als Lernchance** | **Einflussfaktoren auf das Lernverhalten:** | |
|  | **Selbst-Assessment und individuelle Lernzielplanung** | „Weil ich tatsächlich, ähm, also ich hab mir, hatte mir vorgenommen, meine Famulaturen einzugliedern in Innere Medizin, Chirurgie und Notfallmedizin beziehungsweise auch noch Anästhesie und das habe ich vorranging aus strategischen Gründen so gewählt, weil ich, äh, möglichst großen Erfahrungszugewinn in klinischen Grundfähigkeiten erwerben wollte und ich hab mich dann dazu entschieden die Chirurgie zuerst zu machen, [...]" (L1 Zeile 305 - 309) |
|  | **Bewertung der Famulatur anhand der individuellen Lernzielauswahl** | „Also im Prinzip her wars ... gut, dass wir viel in den OP durften, was sicherlich im Vergleich zu anderen Famulaturen durchaus vorteilhaft ist, aber weil ich halt vorher noch gar keine andere Famulatur hatte, ist es, also für die erste Famulatur war es vielleicht nicht sehr passend." (L3 Zeile 22 - 24) |
|  | **Einflussfaktoren auf die Famulaturauswahl** | „Hm, also ich wollte auf jeden Fall in Richtung Innere gehen, weil ich Chirurgie persönlich momentan zumindest nicht so interessant finde […]“ (L6 Zeile 103 - 104) |

| **#3**  **Famulatur als Lernchance**  **(Fortsetzung)** | **Lernprozesse:** | |
| --- | --- | --- |
|  | **Kennenlernen zentraler Prozeduren und Akteure** | „[…] also die ham mir dann, ich durfte nen paar selber, selber CT machen, weil die dann eben gesagt haben, jetzt stellst du am Computer ein und so, dass fand ich schon irgendwie toll, hat mich dann auch gefreut, dachte ich, ach jetzt hab ich hier ein CT gemacht, weils halt ganz schön kompliziert ist und das ist eben in der Radiologie eben auch sehr viel Physik und wie mach ich dann die Ebenen und so und also, […]“ (L7 Zeile 338 - 342) |
|  | **Training von praktischen Fertigkeiten** | „Handwerklich fand ich wichtig, Zugänge legen, Felx, also Flexülenlegen, Blutentnahmen, dass man da auf jeden Fall Routine bekommt, weil das halt Basishandwerk ist." (L2 Zeile 70 - 72) |
|  | **Interaktion mit Patienten** | „[…] äh für mich war der Patientenkontakt und die Gesprächsführung zum Beispiel wichtig, darum hab ich da halt viel Gesprächsführung gemacht, ich bin von den Ärzten allein vorgeschickt worden, hab die ganzen Anamnese gemacht, die klinischen Untersuchungen […] (L4 Zeile 50 - 52) |
|  | **Erhalten eines breiten Einblicks** | „[…] aus dem Grund, weil ich mir dachte, die Herz-Thorax-Chirurgie ist eng assoziiert auch mit internistischen Behandlungen und ich dachte mir, wenn ich jetzt in die Orthopädie gehe, dann sieht die Medikation da meistens so aus, dass nen Antibiotikum und nen Schmerzmittel da reingedonnert wird und da weiß, bei der Herz-Thorax-Chirurgie ist es halt nen bisschen komplexer, also wir sehen halt, äh, der Patienten, die immer noch Risikofaktoren haben, die kriegen Bypässe und dann kriegen sie halt die ganze Palette von Blutverdünnern, Gerinnungshemmern, Cholesterinsenkern, Statinen, zusätzlich dazu dann noch antiinflammatorische Mittel und so weiter und sofort, auch natürlich Antibiotikatherapie postoperativ, wir sehen Wundverschlussstörungen, also es war das die ganze Palette da [...]" (L1 Zeile 313 - 318) |
|  | **Erlernen allgemeiner ärztlicher Routinen** | „Ich hab mir schon was überlegt. Ich wollte unbedingt die internistische Station machen, weil ich erstmal den Stationsalltag allgemein kennenlernen wollte [...]" (L2 Zeile 313 - 315) |
| **#4**  **Famulatur als Lehrchance** | **Tätigkeiten des Lehrens:** | |
|  | **Zeigen** | „[…] Und ähm, dann hat er mir dann quasi nen Modell des Herzens in die Hand gedrückt und hat mir dann an dem Modell gezeigt, hier wir gucken gerade aus der Richtung drauf, sehen da das, da das [...]" (L6 Zeile 76 - 79) |
|  | **Erklären** | „[...] das lief ungefähr so, dass die stellvertretende Chefärztin gemeint hat, ähm, also, die stellvertretende Oberärztin, gemeint hat, gut einer von den Studenten soll direkt mal mit in den OP kommen, weil dann sieht man gleich mal, was hier so gemacht wird. Sehr gut gemacht, hat alles erklärt, was sie, was sie gemacht hat, bei der OP [...]“ (L1 Zeile 78 - 81) |
|  | **Anleiten** | „[...] und dann, äh, hab ich halt öfter mal ne Schwester geholt oder nen Pfleger, Pfleger Roland, war da zum Beispiel, der, der hat mir halt immer mal nen paar Handgriffe gezeigt [...]" (L2 Zeile 138 - 140) |
|  | **Beaufsichtigen** | „Ich durfte mal mit ne Infusion machen, Verbände wechseln durfte ich, Druckverband anlegen, ne Schleuse legen und habe auch 2 Bluttransfusionen gemacht, unter Aufsicht natürlich." (L2 Zeile 72 - 74) |
|  | **Supervidieren** | „[...] ich bin von den Ärzten allein vorgeschickt worden, hab die ganze Anamnese gemacht, die klinischen Untersuchungen [...]. Und hab dann mich quasi mit den Leuten unterhalten und dann kam der Arzt und dann ham wir das zusammen ausgewertet [...]" (L4 Zeile 51 - 55) |
|  | **Beteiligen an ärztlichen Entscheidungsfindungen** | „[...] und dann hat er noch recht spezifische Sachen gefragt und dann hat er noch meine Verdachtsdiagnose gefragt und was wir da geben können [...]" (L4 Zeile 55 - 56) |
|  | **Konzepte der Famulaturausbildung** | |
|  | **Interprofessionelles Lehren** | „[...] gerade im OP die Schwestern wollten da einem auch wirklich viel zeigen und haben auch versucht einen auch son bisschen zu lenken [...]" (L3 Zeile 294 - 295) |
|  | **Initiale Einbindung ins Famulatursetting** | „[...] wir sind angekommen, wurden von der Stationsassistentin erstmal eingewiesen wo was ist, uns wurde die Station gezeigt, also wo man alles findet, uns hat dann nen Pflegehelfer auch erklärt, wie wir an alle Sachen rankommen, die wir auch benötigen und im Prinzip wars so, dass wir am ersten Tag von dem PJtler betreut worden sind." (L1 Zeile 27 - 31) |
|  | **Wissensvermittlung durch Schlüsselcharaktere** | „[…] und immer wenn ich in der Funktionsdiagnostik war, hat sich der Oberarzt sehr viel Mühe gegeben, mir Sachen zu erklären, zum Beispiel im Herzecho, wenn man da nichts erklärt bekommt, dann sieht man da gar nichts.“ (L4 Zeile 73 - 75) |
|  | **Auswirkungen fehlender Famulaturkonzepte** | „Was ich auf jeden Fall nicht so gut fand im Nachhinein, ist, dass ich, ähm, zu viel Blutabnehmen und Flexülen musste, weil naja gut, es musste halt gemacht werden, auf soner Station hat man viele Patienten denen Blut abgenommen werden muss und die Ärzte hatten keine Zeit, beziehungsweise haben sich keine Zeit dafür genommen, auch mal selbst das Blut abzunehmen, so dass ich häufig die ganze Station diesbezüglich versorgen durfte, das dauert dann natürlich ewig." (L6 Zeile 32 - 37) |

| **Supplemental Table 3.** Quantitative survey results from the validation cohort evaluated on a five-level likert scale. Categories and items were derived from qualitative interviews in the  derivation cohort. | | | | | | | | | | |
| --- | --- | --- | --- | --- | --- | --- | --- | --- | --- | --- |
|  | | | | | | | | | |  |
| **Categories and items** | **Responding students, N of 222** | **Response rate** | **Strongy agree** | **Agree** | **Neither agree nor disagree** | **Disagree** | **Strongly disagree** | **Median (25-75 IQR)** | **Mean (SD)** | |
|  |  | **in %** | **N (%)** | **N (%)** | **N (%)** | **N (%)** | **N (%)** |  |  |  |
|  |  |  |  |  |  |  |  |  |  | |
| **Category 1: Coping with insecurities** |  |  |  |  |  |  |  |  |  | |
| 1. I fear, that my skills or my knowledge are not sufficient to persist in the clerkship. | 192 | 86.49 | 21 (10.94) | 48 (25.00) | 62 (32.29) | 27 (14.06) | 34 (17.71) | 3 (2-4) | 3.03 (1.24) | |
| 2. I fear, that the clinical team reacts negatively to insecurities or mistakes from my side. | 190 | 85.59 | 32 (16.84) | 55 (28.95) | 60 (31.58) | 31 (16.32) | 12 (6.32) | 3 (2-3) | 2.66 (1.13) | |
| 3. I fear, that I will not be able to adapt to my new role as a clerkship student. | 193 | 86.94 | 15 (7.77) | 38 (19.69) | 49 (25.39) | 51 (26.42) | 40 (20.73) | 3 (2-3) | 3.33 (1.23) | |
| 4. I fear, that I will be confronted with non-appropriate behaviour by the nursing staff or doctors. | 195 | 87.84 | 15 (7.69) | 39 (20.00) | 44 (22.56) | 64 (32.82) | 33 (16.92) | 3 (2-4) | 3.31 (1.19) | |
| 5. I fear, that I will not be included in the clinical everyday life sufficiently. | 194 | 87.39 | 29 (14.95) | 63 (32.47) | 55 (28.35) | 23 (11.86)) | 24 (12.37) | 3 (2-3) | 2.74 (1.22) | |
|  |  |  |  |  |  |  |  |  |  | |
| **Category 2: The clerkship as a social arrangement** |  |  |  |  |  |  |  |  |  | |
| 6. In the clerkship it is important for me to be part of the doctor's team. | 196 | 88.29 | 100 (51.02) | 77 (39.29) | 15 (7.65) | 4 (2.04) | 0 (0.00) | 1 (1-2) | 1.61 (0.72) | |
| 7. In the clerkship it is important for me to be able to identify with the doctor's team. | 191 | 86.04 | 70 (36.65) | 81 (42.41) | 29 (15.18) | 6 (3.14) | 5 (2.62) | 2 (1-2) | 1.93 (0.94) | |
| 8. In the clerkship it is important for to interact with the other occupational groups in the hospital. | 188 | 84.68 | 54 (28.72) | 81 (43.09) | 47 (25.00) | 6 (3.19) | 0 (0.00) | 2 (1-3) | 2.03 (0.82) | |
| 9. In the clerkship it is important for me, that the different actors in the hospital take time for me. | 193 | 86.94 | 103 (53.37) | 73 (37.82) | 11 (5.70) | 5 (2.59) | 1 (0.52) | 1 (1-2) | 1.59 (0.76) | |
| 10. In the clerkship it is important for me, that I have a good relationship to the other team members. | 189 | 85.14 | 110 (58.20) | 66 (34.92) | 7 (3.70) | 3 (1.59) | 3 (1.59) | 1 (1-2) | 1.53 (0.78) | |
|  |  |  |  |  |  |  |  |  |  | |
| **Category 3: The clerkship as a learning opportunity** |  |  |  |  |  |  |  |  |  | |
| 11. I think, that my pervious knowledge (knowledge/skills) is very important for my learning success in the clerkship. | 202 | 90.99 | 73 (36.14) | 84 (41.58) | 37 (18.32) | 8 (3.96) | 0 (0.00) | 2 (1-2) | 1.9 (0.83) | |
| 12. I think, that my level of training (clinical subjects) is important for my learning success in the clerkship. | 202 | 90.99 | 71 (35.15) | 89 (44.06) | 32 (15.84) | 8 (3.96) | 2 (0.99) | 2 (1-2) | 1.92 (0.87) | |
| 13. I think, that my personal interest in the clinical subject is important for my learning success in the clerkship. | 198 | 89.19 | 123 (62.12) | 61 (30.81) | 9 (4.55) | 5 (2.53) | 0 (0.00) | 1 (1-2) | 1.47 (0.70) | |
| 14. I think, that a preferably broad insight in the clerkship subject is important for my learning success in the clerkship. | 206 | 92.79 | 78 (37.86) | 97 (47.09) | 25 (12.14) | 6 (2.91) | 0 (0.00) | 2 (1-2) | 1.8 (0.76) | |
| 15. I think, that the training of soft skills (e.g. medical consultation) is important for my learning success in the clerkship. | 199 | 89.64 | 50(25.13) | 85 (42.71) | 37 (18.59) | 24 (12.06) | 3 (1.51) | 2 (1-3) | 2.22 (1.01) | |
| 16. I think, that the training are hard skills (e.g. blood sampling) is important for my learning success in the clerkship. | 198 | 89.19 | 111 (56.06) | 60 (30.30) | 21 (10.61) | 6 (3.03) | 0 (0.00) | 1 (1-2) | 1.61 (0.80) | |
| 17. I think, that patient contact is important for my learning success in the clerkship. | 192 | 86.49 | 120 (62.50) | 57 (29.69) | 13 (6.77) | 2 (1.04) | 0 (0.00) | 1 (1-2) | 1.46 (0.67) | |
| 18. I think, that the learning of general medical routines (e.g. patient presentation, visit) is important for my learning success. | 201 | 90.54 | 126 (62.69) | 57 (28.36) | 16 (7.96) | 2 (1.00) | 0 (0.00) | 1 (1-2) | 1.47 (0.69) | |
| 19. I think, that the meeting of central procedures and actors are important for my learning success. | 202 | 90.99 | 74 (36.63) | 96 (47.52) | 31 (15.35) | 1 (0.50) | 0 (0.00) | 2 (1-2) | 1.8 (0.71) | |
| 20. I think, that the setting of own learning goals is important for my learning success. | 203 | 91.44 | 52.00 (25.62) | 84 (41.38) | 53 (26.11) | 14 (6.90) | 0 (0.00) | 2 (1-3) | 2.14 (0.88) | |
|  |  |  |  |  |  |  |  |  |  | |
| **Category 4: The clerkship as a teaching opportunity** |  |  |  |  |  |  |  |  |  | |
| 21. Learning through demonstration and explaining plays for me an important role in the clerkship. | 195 | 87.84 | 133 (68.21) | 53 (27.18) | 8 (4.10) | 1 (0.51) | 0 (0.00) | 1 (1-2) | 1.37 (0.59) | |
| 22. Learning through instruction and supervision plays for me an important role in the clerkship. | 196 | 88.29 | 138 (70.41) | 45 (22.96) | 11 (5.61) | 2 (1.02) | 0 (0.00) | 1 (1-2) | 1.37 (0.64) | |
| 23. Learning through participation in decision-making plays for me an important role in the clerkship. | 194 | 87.39 | 67 (34.54) | 81 (41.75) | 38 (19.59) | 8 (4.12) | 0 (0.00) | 2 (1-2) | 1.93 (0.84) | |
| 24. Interprofessional learning (by nurses, medical technical assistants, etc.) plays for me an important role in the clerkship. | 196 | 88.29 | 65 (33.16) | 76 (38.78) | 42 (21.43) | 11 (5.61) | 2 (1.02) | 2 (1-3) | 2.02 (0.93) | |
| 25. Learning through resource persons (certain nurses, doctors, residents) play for me an important role in the clerkship. | 198 | 89.19 | 115 (58.08) | 62 (31.31) | 16 (8.08) | 5 (2.53) | 0 (0.00) | 1 (1-2) | 1.55 (0.75) | |
| 26. The existence of concrete learning goals is important for clerkships. | 199 | 89.64 | 36 (18.09) | 78 (39.20) | 60 (30.15) | 22 (11.06) | 3 (1.51) | 2 (2-3) | 2.39 (0.96) | |
|  |  |  |  |  |  |  |  |  |  | |
| IQR, inter quartile range; SD, standard deviation; N (%), number and percentage of students having selected the category in relation to total number of students who adressed the item. | | | | | | | | | | |
